# Supplementary figures and images for: A Taxonomic Survey of Female Oviducal Glands in Chondrichthyes: A Comparative Overview of Microanatomy in the Two Reproductive Modes
Source: Animals (Basel). 2021 Sep 9;11(9):2653. doi: 10.3390/ani11092653 (PMC8471067; doi:10.3390/ani11092653)

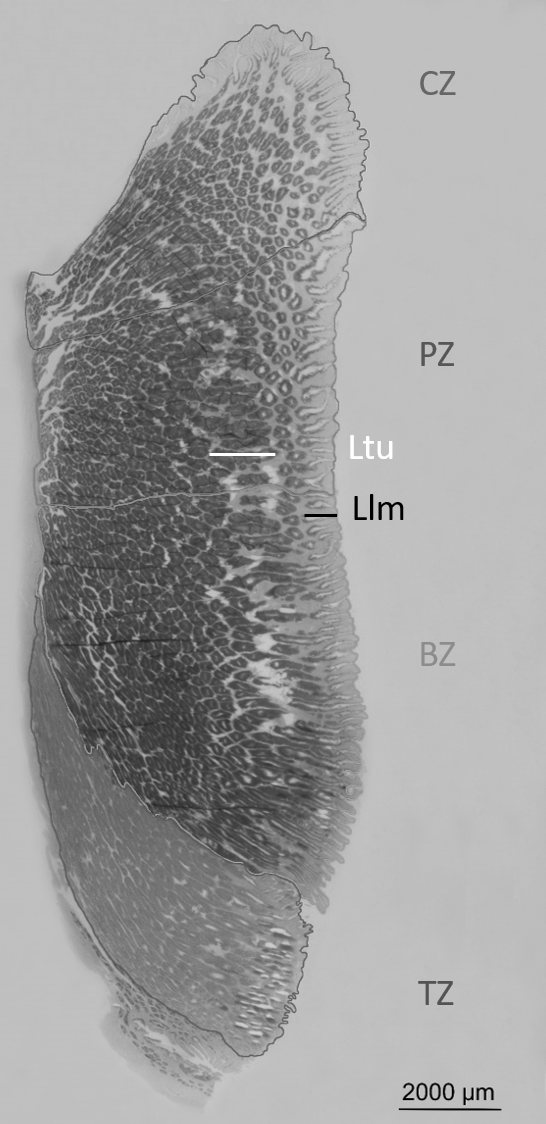

Supplement: Supplementary file 1 [file animals-11-02653-s001.zip › Fig. S1.tif]

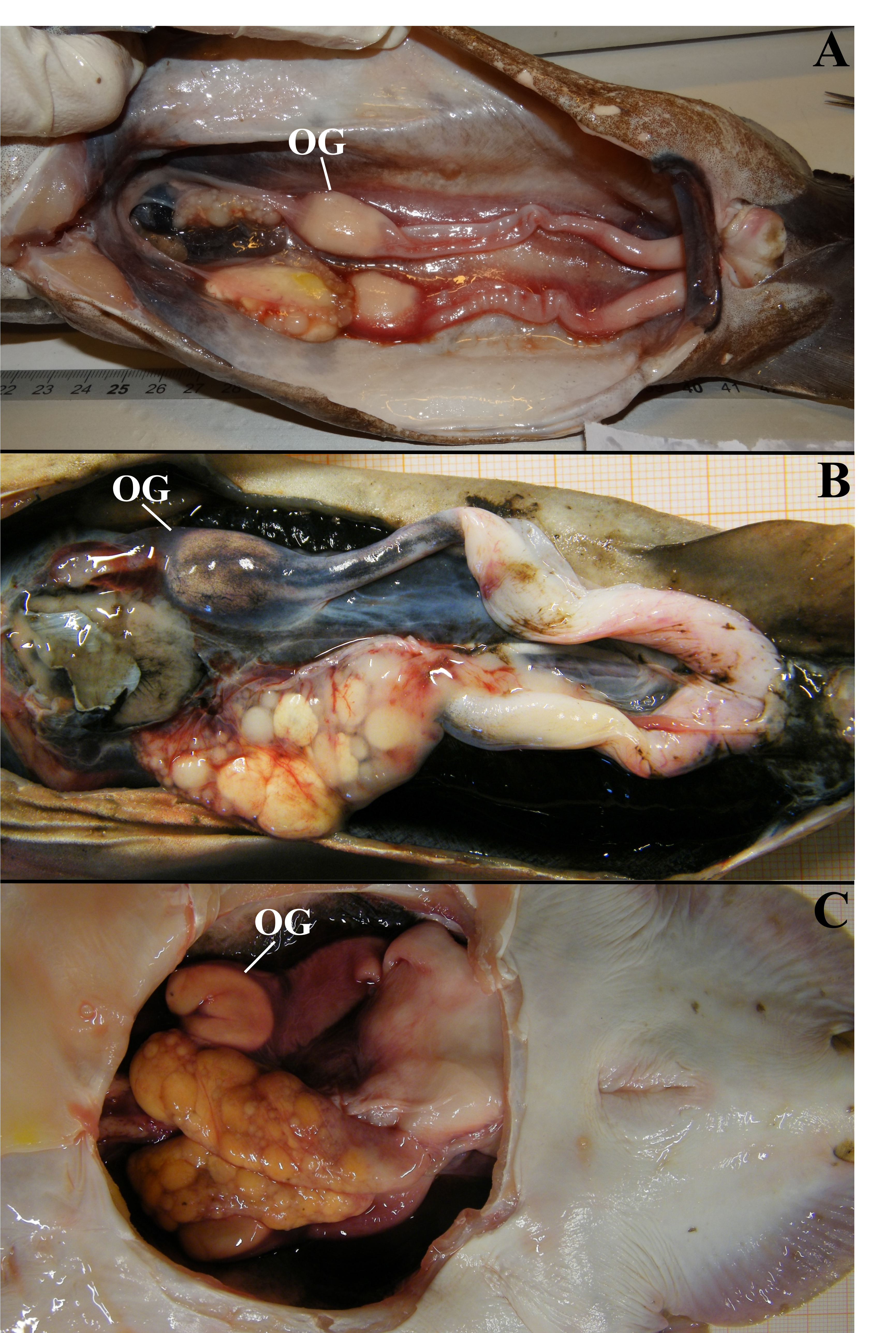

Supplement: Supplementary file 1 [file animals-11-02653-s001.zip › Fig. S2.tif]

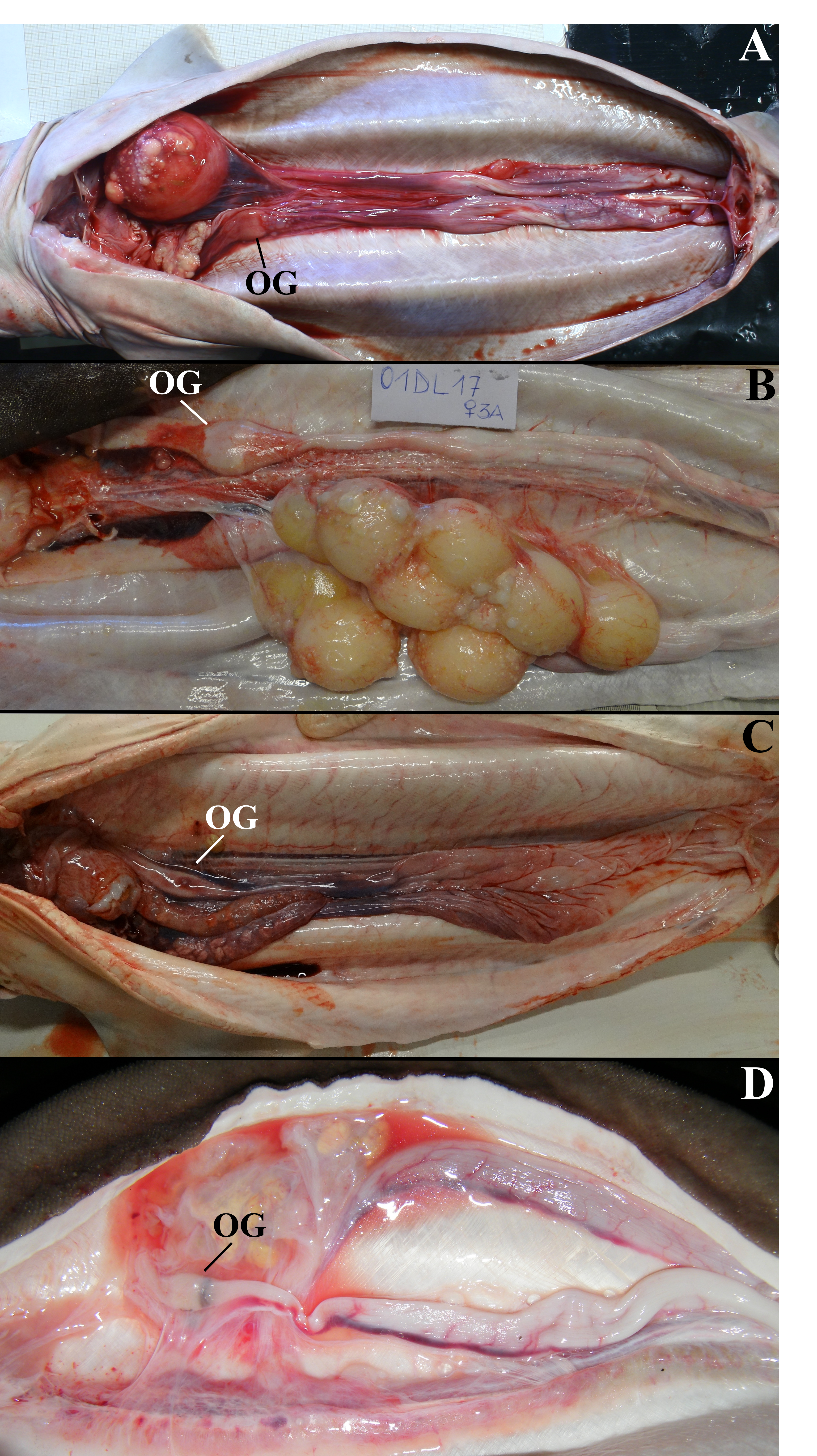

Supplement: Supplementary file 1 [file animals-11-02653-s001.zip › Fig. S3.tif]
